# Atypical fetal junctional ectopic tachycardia: a case report and literature review

**DOI:** 10.1186/s12884-022-04655-6

**Published:** 2022-04-11

**Authors:** Daisuke Katsura, Shunichiro Tsuji, Shinsuke Tokoro, Takako Hoshiyama, Shinsuke Hoshino, Ouki Furukawa, Takashi Murakami

**Affiliations:** 1 Department of Obstetrics and Gynecology, Shiga University of Medical Science Hospital, Setatsukinowa-cho, Otsu, Shiga 520-2192 Japan; 2 Department of Pediatrics, Shiga University of Medical Science Hospital, Otsu, Japan

**Keywords:** Cardiac arrhythmia, Diagnosis, hydrops fetalis, Junctional ectopic tachycardia, Premature restriction of the foramen ovale, Case report

## Abstract

**Background:**

Junctional ectopic tachycardia (JET) is caused by ectopic rhythms, originating in the atrioventricular node, typically with heart rate between 200 and 250 bpm. Herein, we present a case of fetal JET with normal fetal heart rate and a review of nine cases.

**Case presentation:**

A 32-year-old, gravida 2, para 1, woman in whom fetal JET could not be diagnosed prenatally because the fetal heart rate was within the normal range. The fetus was diagnosed with premature restriction of the foramen ovale, and a cesarean section was performed, owing to the right heart overload that was characterized by fetal ascites and abnormal fetal Doppler velocity. Postnatally, the female neonate was diagnosed with JET on a 12-lead electrocardiogram, which revealed a neonatal heart rate of 158 bpm with narrow QRS and atrioventricular dissociation. After failure to respond to amiodarone therapy, she was treated with flecainide, which controlled the JET rate from 120 to 150 bpm. Fetal tachycardia with ventriculo-atrial (VA) dissociation or 1:1 VA conduction with a shorter VA interval than that of atrioventricular reentrant tachycardia confirmed the diagnosis of fetal JET.

**Conclusions:**

JET should be suspected even in the absence of tachycardia in patients with ductus venosus and pulmonary vein retrograde flow or tricuspid and mitral regurgitation without a cardiac anomaly, as tachycardia might sometimes be intermittent in cases of JET.

**Supplementary Information:**

The online version contains supplementary material available at 10.1186/s12884-022-04655-6.

## Background

Fetal arrhythmias are detected in 1–2% of pregnancies. Most fetal arrhythmias are benign and transient; however, persistent fetal arrhythmia can cause low cardiac output and heart failure and lead to fetal hydrops and death. Tachyarrhythmias are diagnosed when the fetal heart rate is persistently > 180 beats per minute (bpm), and they are classified into sinus tachycardia, supraventricular tachycardia, and ventricular tachycardia. Supraventricular tachycardias are divided into atrial tachycardia (atrial flutter and atrial ectopic tachycardia) and conduction system tachycardia (atrioventricular reentrant tachycardia [AVRT], junctional tachycardia, and atrioventricular nodal re-entry tachycardia). Fetal supraventricular tachycardia can be controlled by transplacental administration of anti-arrhythmic drugs such as digoxin, flecainide, sotalol, and, more rarely, amiodarone. Fetal therapy is the recommended management to sufficiently decrease the ventricular rate in order to achieve a good cardiac output for fetuses with hydrops or at high risk of developing hydrops such as those with sustained tachycardia with ventricular rates > 200 bpm [1]. A recent meta-analysis on the transplacental treatment of fetal tachycardia showed that both flecainide and sotalol were more effective than digoxin for conversion of any fetal tachycardia to sinus rhythm, especially in cases of fetal hydrops [2].

Junctional ectopic tachycardia (JET), a rare type of arrhythmia, is caused by ectopic rhythms originating from the atrioventricular (AV) node with a heart rate typically between 200 and 250 bpm, narrow QRS complex, and retrograde atrial conduction in a 1:1 pattern or ventriculo-atrial (VA) dissociation with variable conduction to the atria. Loss of synchronization between the atria and the ventricles disrupts the hemodynamics [3].

Congenital JET is rare and often refractory to medical therapy. It has high morbidity [4], and the causes of death include ventricular fibrillation, AV block, and refractory heart failure with 4–9% mortality rate [5, 6]. One-third of patients with congenital JET have symptoms from the fetal period [7]. JET is associated with high incidence of fetal hydrops despite a relatively low rate of tachycardia [7].

We herein report a case of fetal JET that could not be diagnosed prenatally because the fetal heart rate was within the normal range and present a review of available literature [7–11].

## Case presentation

A 32-year-old, gravida 2, para 1, woman was referred to our hospital at 32 weeks of gestation. During her routine antenatal checkups, frequent premature ventricular contractions were identified before her referral. Her medical and family histories were unremarkable. Fetal ultrasound revealed a maximum vertical pocket of 6.4 cm, an amniotic fluid index of 20 cm, and an mean estimated fetal weight of 2035 ± 1.1 g (standard deviation). Although fetal ascites was noted, no structural anomalies were detected. On fetal Doppler, the umbilical artery pulsatility index was 1.18; the middle cerebral artery pulsatility index and peak systolic velocity were 1.84 and 30.4 cm/s, respectively. Intermittent ductus and pulmonary venosus retrograde flow and pulsatile umbilical venous flow were detected, and the preload index changed from 0.49 to 1.0. The cardiothoracic area ratio was 29.3%, moderate tricuspid and mitral regurgitation was confirmed, the ventricular filling was biphasic–monophasic, and the left and right Tei indexes were 0.44 and 0.52, respectively. On cardiotocography, the fetal heart rate at baseline was 120 bpm with minimal variability; moreover, accelerations and decelerations were not noted. Although premature ventricular contractions were occasionally observed and fetal heart movement had an abnormal impression (Additional file 1), we diagnosed the patient with premature restriction of foramen ovale (PRFO) based on the diameter of the fetal foramen ovale (FO), which was 3 mm. The FO-to-atrial septal length was 0.2, and the right-to-left FO Doppler velocity was 0.5 m/s. A hypermobile atrial septum was detected, and blood flow through the FO was bidirectional. Postnatally, we observed that the abnormal heart movement impression implied AV dissociation.

**Fig. 1:**
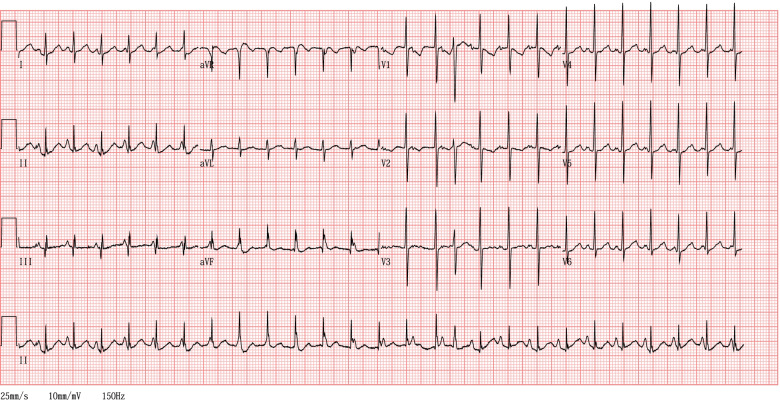
Neonatal 12-lead electrocardiogram. Junctional ectopic tachycardia with narrow QRS and ventriculo-atrial dissociation. The ventricular and atrial rates were 158 and 125 bpm, respectively. The electrocardiogram was recorded at an amplitude of 10 mm/mV and at 25 mm/s

After corticosteroids were administered for fetal lung maturation, cesarean section was indicated due to a right heart overload, which was characterized by fetal ascites and abnormal fetal venous Doppler velocity caused by the PRFO. The female neonate weighed 1956 g, with an Apgar score of 8 and 9 at 1 and 5 min, respectively. Her umbilical arterial blood pH was 7.360. She was admitted into the neonatal intensive care unit for respiratory and circulatory management. In the neonatal intensive care unit, a surfactant was administered for respiratory distress syndrome, and she remained stable with directional positive airway pressure. She was diagnosed with PRFO because an FO diameter of 2 mm was restrictive, and severe tricuspid regurgitation was confirmed. Furthermore, a diagnosis of JET was made because the 12-lead electrocardiogram revealed a neonatal heart rate of 158 bpm with narrow QRS and AV dissociation (Fig. 1).

**Fig. 2:**
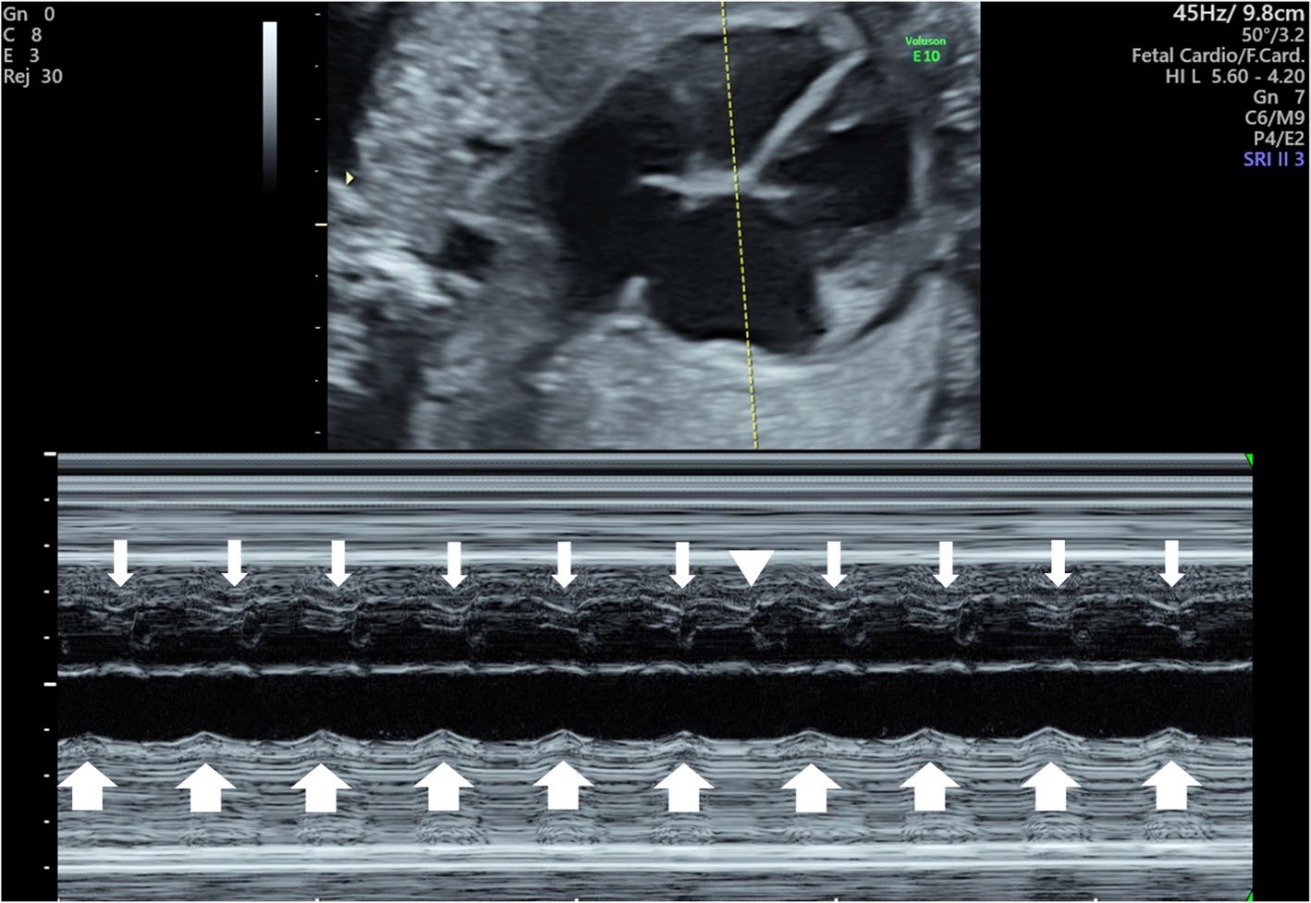
Fetal M-mode ultrasound. Ventriculo-atrial dissociation in atypical junctional ectopic tachycardia; junctional ectopic tachycardia was diagnosed postnatally, and the findings were detected retrospectively. In the four-chamber view of the heart, which shows the left ventricle and right atrium, the wide arrow indicates atrial contraction, the narrow arrow indicates ventricular contraction, and the arrowhead indicates a premature ventricular contraction

Postnatally, the fetal M-mode ultrasound examination revealed AV dissociation (Fig. 2), which could not be noted prenatally. Her heart rate increased to 250 bpm, and amiodarone was administered; however, this was discontinued because of decreasing blood pressure. Thereafter, she was treated with flecainide, which controlled the JET rate from 120 to 150 bpm. The neonate responded well to the treatment.

## Discussion and conclusions

Most fetal arrhythmias are benign and transient, but persistent fetal arrhythmia can cause fetal deterioration. Among them, fetal supraventricular tachycardia often responds to fetal therapy [1].

Fetal JET is a rare type of supraventricular tachycardia often causing severe fetal deterioration, but it is expected to respond to therapy. Therefore, a prenatal diagnosis of JET is important [7]. We encountered a case of fetal JET with a normal fetal heart rate. To the best of our knowledge, fetal JET without tachycardia has never been reported. A summary of all reported cases regarding the clinical course of fetal JET is presented in Table 1 [7–11].

**Table 1:** Comparison of the clinical course of the reported cases of fetal junctional ectopic tachycardia

	Patients								
	1	2	3	4	5	6	7	8	9
Age (years)	32	23	27	34					25
GA at examination (weeks)	32	20	31	19	28	34	23	32	32
FHR (bpm)	130	180	190	200	280	270	180	200	170
1:1 AV conduction		+	+	+	+	+		+	+
AV dissociation	+		–	+			+	+	
Minimal variability	+	+							
DV and PV retrograde flow	+	+	+						+
Fetal hydrops	–	+	+	+	+		+	+	+
Other findings	UV pulsation TR MR	Ventricular filling: monophasic	Ventricular filling: monophasic						UV pulsation
	Ventricular filling: biphasic–monophasic								Dilated RA
	Fetal ascites	fMCG: AV dissociation							TR
									FO abnormally displaced toward the right
Fetal diagnosis	PRFO	JET	JET or VT	JET	JET	JET	JET	JET	JET
Fetal therapy									
Digoxin	–	+ (non-effective)	+ (effective)	+ (non-effective)	+			+ (non-effective)	
Sotalol	–	+ (non-effective)		+ (non-effective)	+			+ (non-effective)	
Amiodarone	–	+ (effective)					+ (effective)		+ (effective)
Flecainide			+ (effective)						
GA at delivery	32	38	37	36	39			36	34
ECG									
HR	150–250	150–170	343						160
1:1 AV conduction			+						+
AV dissociation	+		+						+
Neonatal therapy									
Amiodarone	+ (stopped because of side effect)	–		+ (effective)				+ (effective)	+ (effective)
Flecainide	+ (effective)		+ (effective)						
Propranolol		+ (effective)	+ (effective)						
Prognosis	JET under control	Sinus rhythm	JET under control	Sinus rhythm	Stop postnatally	Stop prenatally		Sinus rhythm	JET under control

*AV* atrioventricular, *DV* ductus vein, *ECG* electrocardiogram, *FHR* fetal heart rate, *fMCG* fetal magnetocardiography, *FO* foramen ovale, *GA* gestational age, *JET* junctional ectopic tachycardia, *MR* mitral regurgitation, *PRFO* premature restriction of foramen ovale, *PV* pulmonary vein, *RA* right atrium, *TR* tricuspid regurgitation, *UV* umbilical vein, *VT* ventricular tachycardia; + = present, − = absent, blank = not described

Patient no. (reference): 1, our report; 2, Zaidi et al. [7]; 3, Zaidi et al. [7]; 4, Fouron [10]; 5, Fouron [10]; 6, Fouron [10]; 7, Strasburger et al. [11]; 8, Fouron et al. [9]; 9, Lupoglazoff et al. [8]

In previous reports, the mean maternal age was 28.2 years (range, 23–34 years), and the mean gestational age at diagnosis was 27.8 weeks (range, 19–34 weeks). None of the cases had cardiac anomaly. All except our case had tachycardia, and the mean fetal heart rate was 208.7 bpm (range, 170–280 bpm). JET presents as tachycardia with minimal heart rate variability and VA dissociation or 1:1 VA conduction [7]. The VA dissociation supports the diagnosis of JET. Regarding the 1:1 VA conduction, JET presents with a short VA interval; JET (close to 0) has a shorter VA interval than AVRT; hence, it should be distinguished from AVRT [5]. All cases of 1:1 VA conduction had tachycardia with simultaneous onset of atrial and ventricular contractions; this is useful for the diagnosis of JET. Minimal heart rate variability was detected in cases 1 and 2; it was not described in the other cases. In most of the cases, the patients had effusion into the fetal body cavities, including fetal hydrops (77.7%, 7/9) and fetal ascites (22.3%, 1/9). At diagnosis, ductus and pulmonary venosus retrograde flow, umbilical vein pulsation, tricuspid and mitral regurgitation, and monophasic ventricular filling were detected. The monophasic ventricular filling cycle might be due to the fusion of the filling of the ventricles during generalized diastole (E wave) and the active filling of the ventricles during atrial systole (A wave) caused by tachycardia; however, these fetal Doppler findings suggest that VA dissociation or 1:1 VA conduction impairs fetal ventricular filling and increases atrial pressure. In our case, these findings were intermittent, which might have changed the effect on the ventricular filling. Additionally, increased atrial pressure resulted in the PRFO, and similar findings were detected in case 9. Unfortunately, we could not diagnose JET prenatally because we did not notice a VA dissociation, as the fetal heart rate was normal; although VA dissociation was detected on fetal M-mode ultrasound examination retrospectively. Therefore, a cesarean section was performed, considering the worsening of the right heart overload due to the PRFO. Although our patient had a normal fetal heart rate and the possibility of junctional rhythm was considered, she might have had intermittent tachycardia because signs of fetal hemodynamic deterioration, such as fetal ascites, ductus and pulmonary venosus retrograde flow, pulsatile umbilical venous flow, and tricuspid and mitral regurgitation, were present. In case 3, the JET and ventricular tachycardia could not be differentiated. The JET was diagnosed by fetal magnetocardiography (fMCG) in case 2 and by ultrasonography in other cases. fMCG is effective for diagnosing fetal arrhythmias; however, it cannot be performed in all facilities. Therefore, ultrasonographic findings are important for diagnosing fetal JET. Regarding prognosis, in case 5, the effect of sotalol was not mentioned, but the JET naturally improved postnatally. In cases 4 and 8, sotalol and digoxin had no effect; therefore, an emergency cesarean section was performed, as the fetal hydrops worsened. However, both neonates responded to amiodarone postnatally, attaining sinus rhythm. All fetuses who received prenatal flecainide or amiodarone responded to the therapy. The JET changed to sinus rhythm in case 2, fetal hydrops improved in cases 3 and 7, and the fetal heart rate returned to the normal range in case 9. Our patient was delivered at 32 weeks of gestation, whereas all patients who received fetal therapy with flecainide or amiodarone were delivered after 34 weeks of gestation. Although our patient was delivered at 32 weeks of gestation, no indication for fetal treatment was noted—even though we could diagnose JET prenatally, as the fetal heart rate was within the normal range on cardiotocography. In case 9, the mother had a delivery history of a neonate with JET. A familial association with JET was noted in 20–50% of patients [5, 12]. Thus, in a pregnant woman presenting with fetal JET, a detailed medical and family history is required.

In neonates with JET, amiodarone is used as a first-line agent, either alone or in combination with propranolol or flecainide [3]. Administration of antiarrhythmic drugs in pregnant women with fetal tachyarrhythmias improved the sinus rhythm of their fetuses [13]. Among the antiarrhythmic drugs, flecainide and amiodarone, which are effective for fetal JET, have been used as second-line agents [13]. Although fetal death has been previously attributed to flecainide [14], recent reports have shown its safety for mother and fetus as well as its efficacy for fetal tachycardia [13, 15, 16]. Amiodarone has a more significant toxicity profile, such as maternal and neonatal AV block and QT prolongation with neonatal thyroid dysfunction [11]; however, it has been used to successfully treat fetal supraventricular tachycardia when other multiple antiarrhythmic drugs fail [11]. Therefore, in fetuses with JET, flecainide might be the initial drug of choice; however, amiodarone should be considered if flecainide fails. Furthermore, careful maternal and fetal monitoring is required during treatment because both drugs have maternal and fetal side effects.

In conclusion, fetal tachycardia with VA dissociation or 1:1 VA conduction with a shorter VA interval (close to the simultaneous onset of atrial and ventricular contractions) than that of AVRT supports the diagnosis of fetal JET. JET should be diagnosed prenatally, and treatment with antiarrhythmic drugs should be considered in pregnant women to improve fetal condition and prevent preterm birth. Additionally, JET should be considered a differential diagnosis even in the absence of tachycardia in patients with ductus venosus and pulmonary vein retrograde flow or tricuspid and mitral regurgitation without a cardiac anomaly, as tachycardia might sometimes be intermittent in cases of JET.

## Supplementary Information

**Additional file 1:** Video of fetal ultrasound. A four-chamber view showing the heart movement with ventriculo-atrial dissociation in junctional ectopic tachycardia.

## Data Availability

The datasets used and/or analysed during the current study available from the corresponding author on reasonable request.

## Abbreviations

AV: Atrioventricular
fMCG: Fetal magnetocardiography
JET: Junctional ectopic tachycardia
PRFO: Premature restriction of foramen ovale
VA: Ventriculoatrial
FO: Foramen ovale
AVRT: AV reentrant tachycardia
